# Adherence of Mobile App-Based Surveys and Comparison With Traditional Surveys: eCohort Study

**DOI:** 10.2196/24773

**Published:** 2021-01-20

**Authors:** Chathurangi H Pathiravasan, Yuankai Zhang, Ludovic Trinquart, Emelia J Benjamin, Belinda Borrelli, David D McManus, Vik Kheterpal, Honghuang Lin, Mayank Sardana, Michael M Hammond, Nicole L Spartano, Amy L Dunn, Eric Schramm, Christopher Nowak, Emily S Manders, Hongshan Liu, Jelena Kornej, Chunyu Liu, Joanne M Murabito

**Affiliations:** 1 Department of Biostatistics Boston University School of Public Health Boston, MA United States; 2 Section of Preventive Medicine and Epidemiology and Cardiovascular Medicine, Department of Medicine, and Department of Epidemiology Boston University Schools of Medicine and Public Health Boston, MA United States; 3 Boston University's and National Heart, Lung, and Blood Institute's Framingham Heart Study Framingham, MA United States; 4 Center for Behavioral Science Research Department of Health Policy & Health Services Research Boston University Henry M Goldman School of Dental Medicine Boston, MA United States; 5 Cardiology Division Department of Medicine University of Massachusetts Medical School Worcester, MA United States; 6 Department of Quantitative Health Sciences University of Massachusetts Medical School Worcester, MA United States; 7 Care Evolution Ann Arbor, MI United States; 8 Section of Computational Biomedicine Department of Medicine Boston University School of Medicine Boston, MA United States; 9 Cardiology Division Department of Medicine University of California San Francisco San Francisco, CA United States; 10 Section of Endocrinology, Diabetes, Nutrition, and Weight Management Boston University School of Medicine Boston, MA United States; 11 Section of General Internal Medicine Department of Medicine Boston University School of Medicine Boston, MA United States

**Keywords:** eCohort, mobile health, mHealth, smartphone, survey, app, Framingham Heart Study, adherence, agreement, cardiovascular disease

## Abstract

**Background:**

eCohort studies offer an efficient approach for data collection. However, eCohort studies are challenged by volunteer bias and low adherence. We designed an eCohort embedded in the Framingham Heart Study (eFHS) to address these challenges and to compare the digital data to traditional data collection.

**Objective:**

The aim of this study was to evaluate adherence of the eFHS app-based surveys deployed at baseline (time of enrollment in the eCohort) and every 3 months up to 1 year, and to compare baseline digital surveys with surveys collected at the research center.

**Methods:**

We defined adherence rates as the proportion of participants who completed at least one survey at a given 3-month period and computed adherence rates for each 3-month period. To evaluate agreement, we compared several baseline measures obtained in the eFHS app survey to those obtained at the in-person research center exam using the concordance correlation coefficient (CCC).

**Results:**

Among the 1948 eFHS participants (mean age 53, SD 9 years; 57% women), we found high adherence to baseline surveys (89%) and a decrease in adherence over time (58% at 3 months, 52% at 6 months, 41% at 9 months, and 40% at 12 months). eFHS participants who returned surveys were more likely to be women (adjusted odds ratio [aOR] 1.58, 95% CI 1.18-2.11) and less likely to be smokers (aOR 0.53, 95% CI 0.32-0.90). Compared to in-person exam data, we observed moderate agreement for baseline app-based surveys of the Physical Activity Index (mean difference 2.27, CCC=0.56), and high agreement for average drinks per week (mean difference 0.54, CCC=0.82) and depressive symptoms scores (mean difference 0.03, CCC=0.77).

**Conclusions:**

We observed that eFHS participants had a high survey return at baseline and each 3-month survey period over the 12 months of follow up. We observed moderate to high agreement between digital and research center measures for several types of surveys, including physical activity, depressive symptoms, and alcohol use. Thus, this digital data collection mechanism is a promising tool to collect data related to cardiovascular disease and its risk factors.

## Introduction

eCohorts use new sensor devices and smartphone technology for longitudinal research data collection [1]. Technology permits the identification of digital biomarkers of health and improvement in health-related behaviors [2-5]. Mobile apps may be a promising and feasible tool for health interventions [6,7], and most previous studies have shown that mobile health (mHealth) plays an important role in promoting behavior change for children, adolescents, and young adults [8,9]. However, eCohorts are challenged by low adherence [10,11] and may yield substantial volunteer bias [12], raising concerns about the generalizability of study findings. For example, in the MyHeart Counts Cardiovascular Health Study [11] that examined the feasibility of a smartphone-based assessment of physical activity, less than 10% of enrolled participants completed the full 7 days of physical activity data. Because of low adherence rates and sampling bias, it remains unclear how these previous study results will reflect the whole community.

Establishing the validity and reliability of new electronic data collection methods is required before deploying digital technology in epidemiology settings [13]. A few studies have investigated the equivalence of questionnaires administered on different electronic devices versus traditional paper data collection [14-17]. The electronic modes in these studies included a tablet, touchscreen, interactive voice response system, and personal digital assistant. A few studies attempted to create smartphone apps for specific clinical use, which demonstrated scientific validity [18,19]. However, reliability assessment of mobile app surveys remains scarce, especially for cardiovascular phenotypes.

To integrate digital and mHealth data into a traditional longitudinal cohort, we leveraged an in-person examination as part of the Framingham Heart Study (FHS) to enroll participants into an eCohort (eFHS) using a new smartphone app, digital blood pressure cuff, and smartwatch [20]. Embedding our eCohort in the FHS allowed us to compare the digital measures obtained from a smartphone with the same surveys obtained during in-person clinical examinations at the FHS Research Center using research protocols [21]. To that end, the objective of this study was threefold: (1) to determine the app-based survey adherence over a 12-month period, (2) to compare baseline eFHS app survey measurements to research center measures, and (3) to examine the association of periodic app-based survey measures across different time points. We hypothesized that embedding eFHS in the FHS and leveraging the in-person exam to enroll participants would result in high app-based survey adherence at baseline that would decrease over the 1-year follow-up period. We also hypothesized that app-based surveys would be comparable with surveys collected at the research center.

## Methods

### Study Sample

Participants of the FHS Third Generation (Gen 3) cohort (n=4095), Omni Group 2 (n=410), and New Offspring Spouse (n=103) were recruited from 2002 to 2005, and underwent periodic research examinations every 6 to 8 years [21]. We leveraged exam 3 (2016 to 2019) to invite English-speaking FHS participants who owned a smartphone (including iPhone 4S or higher with at least iOS 8.2, or an Android phone as of October 30, 2017) to enroll in the eFHS. The eFHS study was approved by the Institutional Review Board at Boston University Medical Center. Beginning in June 2016, at the time of the in-person research examination, participants were invited to download the eFHS smartphone app from the Apple App Store. The participants were not required to register and complete the app-based surveys at baseline at the research center. Some participants chose to register after leaving the research center. Participation in the eFHS was voluntary and participants were not incentivized for participating. All participants were provided with a written protocol that includes information of how to download the app, enter registration information, sign the electronic consent form, and enable notifications on the phone (Multimedia Appendix 1). Participants reached the first screen upon logging into the app after registration. The list of surveys was organized by due date and displayed on the survey screen (Multimedia Appendix 1). Participants received different types of notifications such as welcome messages on enrollment to the study, notifications when new surveys became available, reminder notifications to complete surveys, and thank you messages after completing all surveys (Multimedia Appendix 2). Collected survey data in this study were pushed to a secure cloud server and transmitted to FHS Research Center servers. Among the 3521 participants (Multimedia Appendix 3) who came to the research center (from April 2016 to April 2019), we excluded 1370 participants who were ineligible (did not own a smartphone), did not consent (owned a smartphone but declined participation), or had incompatible phones, and those who enrolled in eFHS but had less than a 12-month follow-up period (n=203).

### eFHS Smartphone App

Health surveys were distributed at baseline (app download) and at 3-month intervals for 1 year [20] to collect sociodemographic data and variables related to cardiovascular disease (CVD). Corresponding surveys were administered in the research center by trained research technicians or physician/nurse practitioners, except for the sociodemographic survey and health survey questions, which were self-administered. A total of 22 surveys were deployed on the smartphone over the 12-month period after enrollment. Nine baseline surveys for self-reported data were deployed in the following order: sociodemographic information, smoking, medications and self-reported risk factors, baseline CVD history, baseline non-CVD medical history, physical activity, alcohol consumption, health survey, and depressive symptoms (assessed with the Center for Epidemiologic-Studies Depression Scale [CES-D]) [22]. Among the nine baseline surveys, the physical activity survey was deployed at each 3-month interval after registration. A medical history update, depressive symptoms (CES-D), and health survey were gathered at 6 months and 12 months. Surveys for medication use, self-reported risk factors, and smoking and alcohol consumption were collected at 12 months after the baseline survey. A short description of each survey (Multimedia Appendix 4) and the number of steps to complete each survey were provided to the participants. The first step provided the purpose of the survey, intermediate steps contained one or more survey questions, and the last step thanked participants for completing the survey (see Multimedia Appendix 1 for screenshots of some steps of the Physical Activity Index [PAI] survey). Detailed descriptions of screens or steps of each of 10 different eFHS app surveys can be found in our previous report [20].

### eFHS Smartphone Survey 3-Month Interval

**Figure 1:**
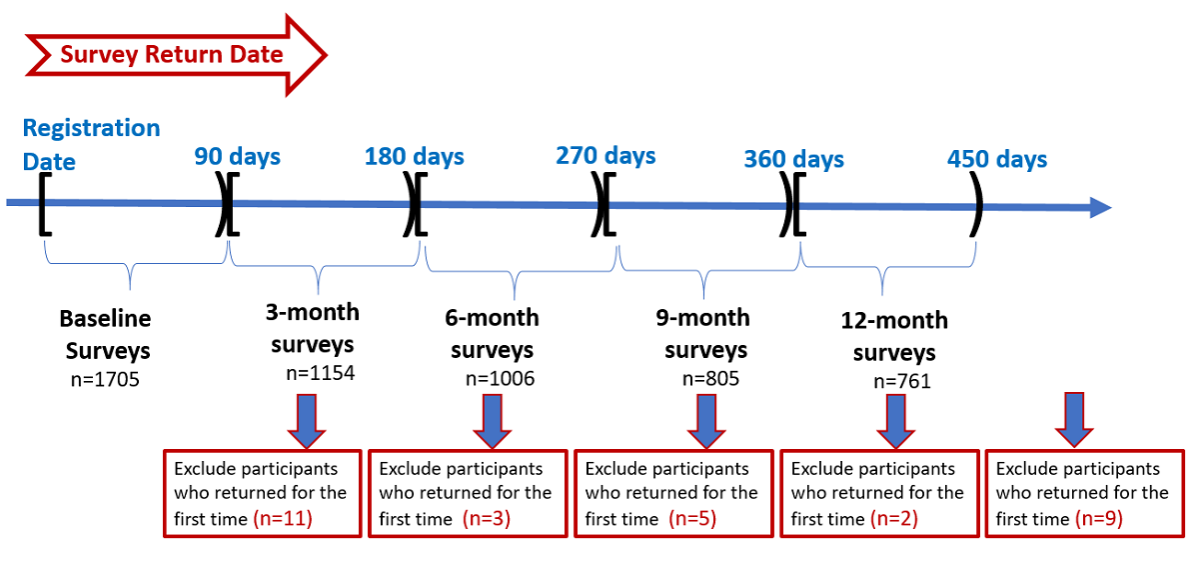
The time window approach for separating baseline and other follow-up surveys (here, completing a survey means completing 75% of questions in the given survey).

We evaluated adherence by assessing the return of baseline survey data within 90 days of registration. To distinguish returned survey data from baseline or additional follow-up surveys, we used a time window approach (Figure 1). Thirty participants were excluded because they returned surveys for the first time after 90 days from the registration. We considered the survey data as baseline surveys if participants returned data between 0 to 89 days from registration. If the surveys were returned between 90 to 179 days from the registration date, we considered the data as 3-month surveys. Similarly, we considered surveys as 6-month, 9-month, and 12-month survey data if participants returned their surveys within 180-269 days, 270-359 days, and 360-449 days from the registration dates, respectively.

### Survey Return Time and Touch Time

To evaluate survey completion time, we examined the survey return time and touch time. The survey return time was calculated by considering the time between the deployment of the survey and the return of a survey. The touch time was calculated by taking the time between the start and return of a survey. We computed the median and IQR of the touch time and survey return time for each type of survey (Multimedia Appendix 5). Furthermore, we calculated the step time (time taken to complete each step) and question time (time taken to complete a question).

### Survey Adherence

To minimize frustration, we allowed eFHS participants to skip questions in a given survey. We defined survey completion (completing one survey) if a participant completed 75% of all questions in a given survey (Multimedia Appendix 6). We used two methods for calculating survey adherence. We first calculated the proportion of individuals who completed at least one survey at a given 3-month time window. The second method calculated the proportion of individuals who completed all surveys at a given 3-month time window (Multimedia Appendix 7).

### Statistical Analysis

We compared the characteristics of FHS participants who provided consent for eFHS with those of participants who declined or were not eligible for this study. All characteristic variables were collected at research center health examination 3. We used Student *t* tests for continuous variables and χ^2^ tests for categorical variables. Among the participants who enrolled in eFHS, we compared the characteristics of participants who returned smartphone app surveys with those of participants who did not return surveys. We used a multivariable (adjusted) logistic regression model, which included an indicator variable (to denote the eFHS participants who returned surveys and those who did not return) as the dependent variable adjusting for age, sex, current smoking, and highest education level.

Surveys collected at the research center health examination and the eFHS app-based surveys provided two sets of measurements for sociodemographic and medical information. To evaluate the agreement of digital survey measures, we compared surveys at eFHS baseline to surveys collected from research center examination 3 using the concordance correlation coefficient (CCC) [23,24] and Bland-Altman plots [25,26]. We used the measurements in the research center as the gold standard for the Bland-Altman analysis. We investigated three different types of surveys deployed at different time intervals: PAI, depressive symptoms surveys (CES-D score), and alcohol consumption surveys. The PAI surveys were deployed every 3 months, CES-D surveys were deployed every 6 months, and the alcohol surveys were deployed at baseline and at 12 months. The surveys collect different health behaviors and mood information that might be reported differently when administered by a trained examiner vs self-reported on a smartphone app.

We calculated PAI as a weighted composite score [27-29] of activity levels with corresponding weights of 1 (for sleep), 1.1 (for sedentary), 1.5 (for slight), 2.4 (for moderate), and 5 (for heavy activities). The number of hours for each physical activity variable (sleep, sedentary, slight, moderate, and heavy) summed to 24 hours. If an individual had one variable missing, we imputed the missing value with 24 minus the sum of hours from the other four variables (n=77).

We analyzed depressive symptoms with two variables: a continuous variable that was the summation of the individual CES-D scores and a binary variable that was defined as 1 if the sum of the CES-D score was ≥16 and 0 otherwise [30,31]. To calculate the continuous CES-D variable, we considered only participants who answered all 20 questions. We found that 72 participants skipped at least one question. We imputed the 61 missing values using the following rule [32]: if more than 5 items were missing, all observations were considered as missing (n=11); if 1 to 5 items were missing, then the average value of the nonmissing items was multiplied by 20.

For alcohol use, we used average drinks per week calculated by the number of drinks per day times the number of days a participant had any type of alcoholic beverage per week.

We used the CCC for continuous measures and the Cohen κ coefficient [33,34] for categorical predictors for agreement analysis between measures from the same participants between eFHS app surveys and research center questionnaires.

We also examined whether three measures (PAI, the sum of CES-D, and average drinks per week) displayed any trend across time points. We used linear mixed models to compare means of PAI and depression symptom measures at each 3-month period. We used the paired *t* test to compare alcohol consumption at baseline and 12 months. We used the R program (version 3.6.1) for all statistical analyses and considered two-sided *P* values <.05 to indicate statistical significance.

## Results

### Study Sample and Survey Metrics

We included 1948 eFHS participants who met the eligibility criteria and had a follow-up time of 12 months or longer (Multimedia Appendix 3) for analysis. Compared to FHS participants who were not enrolled in eFHS, enrolled participants were more likely to be women, white, married, employed full time, completed bachelor or higher degrees, report excellent health, and have more favorable CVD risk factor levels (Table 1). Among the enrolled eFHS participants, 1735 participants returned surveys. Participants who returned surveys were more likely to be women (adjusted odds ratio [aOR] 1.58, 95% CI 1.18-2.11) and were less likely to be current smokers (Multimedia Appendix 8).

**Table 1:** Characteristics of participants in the eCohort Framingham Heart Study (eFHS) and Framingham Heart Study (FHS) participants not enrolled in eFHS.^a^

Variable		eFHS participants (n=1948)	FHS participants not enrolled in eFHS (n=1566)	*P* value
Age (years), mean (SD)		52.8 (8.7)	56.6 (9.8)	<.001
Female sex, n (%)		1109 (56.93)	782 (49.94)	<.001
**Race, n (%)**				.003
	White	1813 (93.07)	1414 (90.29)	
	Black	30 (1.54)	29 (1.85)	
	Hispanic	45 (2.31)	60 (3.83)	
	Asian	28 (1.44)	43 (2.75)	
	Other	32 (1.64)	20 (1.28)	
BMI (kg/m^2^), median (IQR)		27.3 (24.3-31.3)	28.2 (24.6-32.6)	<.001
Systolic blood pressure (mmHg), mean (SD)		119 (14)	121 (15)	<.001
Diastolic blood pressure (mmHg), mean (SD)		76 (8)	76 (9)	.33
Current smoking, n (%)^b^		108 (5.55)	125 (7.99)	.005
Diabetes mellitus, n (%)^c^		122 (6.29)	189 (12.36)	<.001
Hypertension, n (%)^d^		511 (26.26)	582 (37.26)	<.001
Atrial fibrillation, n (%)		36 (1.85)	52 (3.32)	.008
Prevalent cardiovascular disease, n (%)		67 (3.44)	97 (6.19)	<.001
Physical Activity Index score, median (IQR)		32.60 (30.10-35.50)	33 (30.20-36.40)	.004
**Highest education level achieved, n (%)**				<.001
	Less than high school	13 (0.67)	35 (2.26)	
	Completed high school	168 (8.66)	301 (19.46)	
	Completed some college	467 (24.08)	441 (28.51)	
	Bachelor’s degree	741 (38.22)	479 (30.96)	
	Graduate or professional degree	550 (28.37)	291 (18.81)	
Married, living as married, living with partner, n (%)^e^		1446 (74.73)	1002 (65.15)	<.001
Self-reported health as excellent, n (%)^f^		1414 (72.62)	913 (58.60)	<.001
Employed full time, n (%)^g^		1361 (70.19)	913 (59.32)	<.001

^a^Data reflect enrollment up to January 28, 2019.

^b^eFHS n=1947, FHS n=1564.

^c^eFHS n=1940, FHS n=1529.

^d^eFHS n=1946, FHS n=1562.

^e^eFHS n=1935, FHS n=1538.

^f^eFHS n=1947, FHS n=1558.

^g^eFHS n=1939, FHS n=1539.

There were 1705 participants who returned baseline surveys within the 3-month interval (Multimedia Appendix 3). Survey return time, touch time, step time, and time spent for each question for each survey at each survey wave are presented in Multimedia Appendix 5. Participants returned baseline and follow-up surveys within 2 weeks with the exception of the 12-month medical history update that was returned within 30 days (Multimedia Appendix 9). The actual time taken to complete surveys (touch time) was less than 5 minutes.

The number of participants who returned a specific type of survey at each survey wave is displayed in Multimedia Appendix 10. Most participants completed 75% of the questions of at least one baseline survey (1704/1918, 88.84%; see Multimedia Appendix 7). Participants continued to return some surveys at 12 months (757/1918, 39.47%; see Multimedia Appendix 7) but only a few participants (28/1918, 1.46%; see Multimedia Appendix 10) completed the 12-month medical history update questionnaire.

### Survey Adherence/Completion

**Figure 2:**
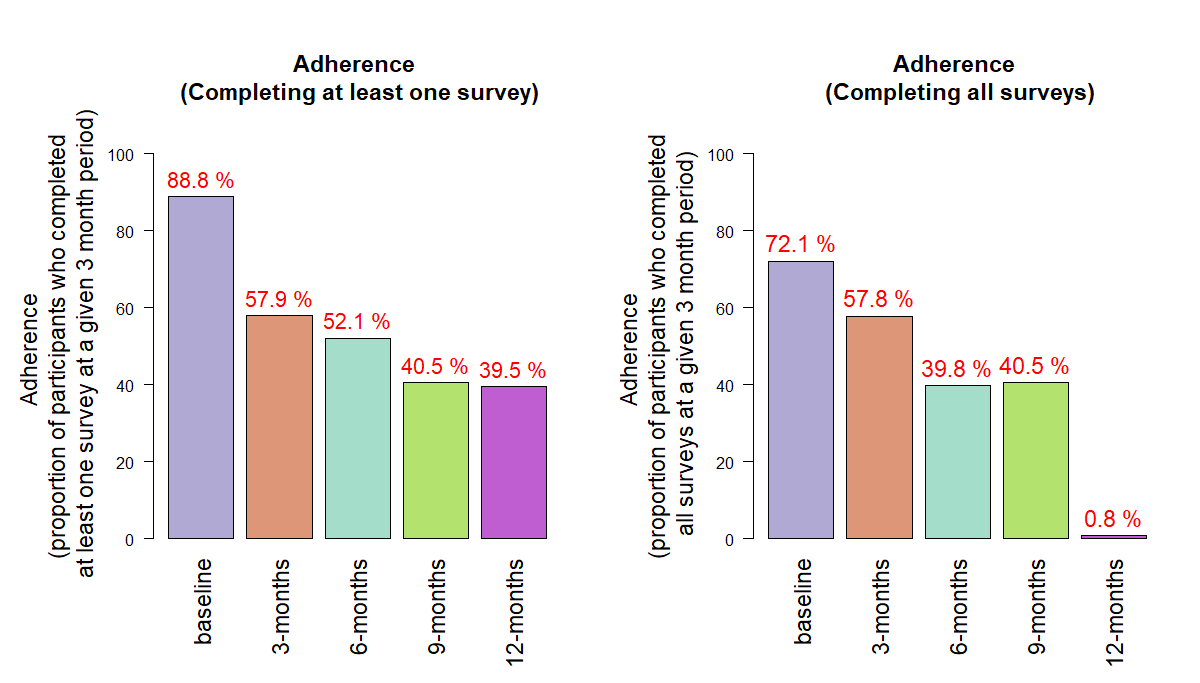
Proportion of eCohort Framingham Heart Study (eFHS) participants who completed at least one survey (left panel) and all surveys (right panel).

Among participants who returned surveys, more than 85% completed more than 75% of the questions across all surveys at all time windows (Multimedia Appendix 6). Considering the proportion of individuals who completed at least one survey at a given 3-month window (Multimedia Appendix 7), eFHS participants had the highest adherence at baseline (89%) and the adherence decreased over time (58% at 3 months, 52% at 6 months, 41% at 9 months, and 40% at 12 months) (Figure 2). We observed similar adherence rates based on the proportion of participants who completed all surveys at a given 3-month period (72% at baseline, 58% at 3 months, 40% at 6 months, 41% at 9 months). At 12 months, adherence was reduced to 1% because most participants did not return the 12-month medical history update survey.

### Comparison of Baseline eFHS App Survey With Research Center Measurements

**Figure 3:**
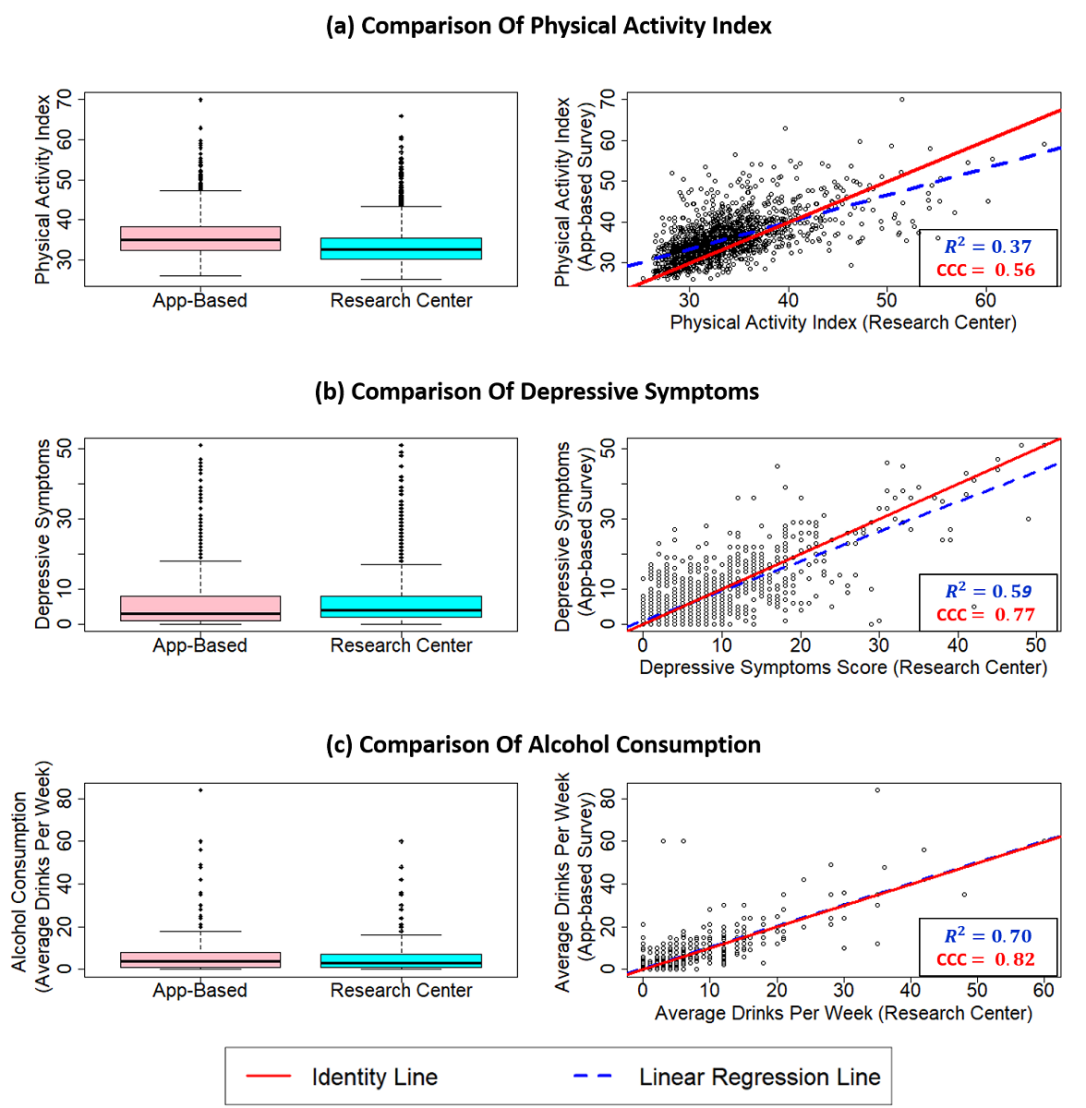
Comparison of baseline surveys: Physical Activity Index (PAI), depression symptoms scale (Sum of Center For Epidemiologic-Studies Depression Scale [CES-D] scores), and alcohol consumption (average drinks per week).

The baseline app-based survey data for PAI (n=1545), CES-D score (n=1628), and average alcohol consumption per week (n=1513) were used to compute the mean difference between the mobile app surveys and the questionnaires collected in the research center. The mobile app survey had a higher PAI compared to the respective in-research center exam (mean difference 2.27). We observed high agreement (CCC=0.82, 95% CI 0.81-0.84) between the two alcohol consumption measures and the two CES-D scores (CCC=0.77, 95% CI 0.75-0.79) (Figure 3). Moderate agreement was observed between the two PAI measures (CCC=0.56, 95% CI 0.52-0.59) and the binary depression variables (Cohen κ=0.51, 95% CI 0.44-0.58). The Bland-Altman plots of PAI, CES-D scores, and alcohol consumption showed that the spread of the difference increased with increasing mean of the observations (bias increased and variability was not consistent across the graph), likely reflecting that the distributions were skewed (Multimedia Appendix 11).

### Association of Periodic App-Based Survey Measures Across Different Time Points

**Figure 4:**
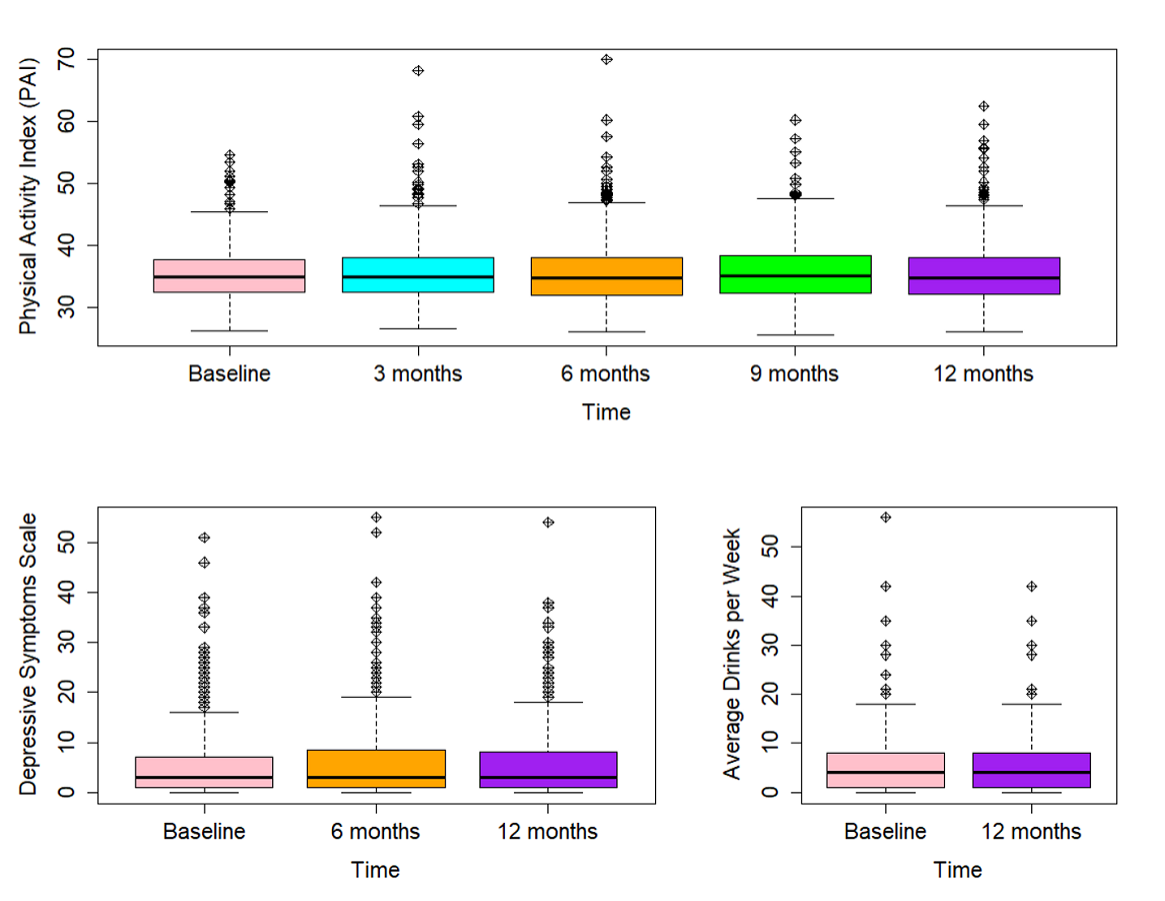
Boxplots for Physical Activity Index (PAI), depressive symptoms scale (Center For Epidemiologic-Studies Depression Scale [CES-D] score), and average drinks per week across different time points.

Overall, 539 participants returned physical activity questionnaires at all five time points (baseline, 3 months, 6 months, 9 months, and 12 months), 644 participants returned depressive symptom surveys at all three time points (baseline, 6 months, and 12 months), and 613 participants returned all alcohol consumption surveys (baseline and 12 months). PAI (for all time effects compared to baseline) and alcohol consumption (mean difference of baseline to 12-month questionnaire –0.03, *P*=.82) were at similar levels across several time points, whereas the CES-D score slightly increased from baseline to 6 months (slope=1.01, *P*<.001) and from baseline to 12 months (slope=0.84, *P*<.001) (Figure 4).

## Discussion

### Principal Results

Our findings in this middle-aged community-based sample are threefold. First, eFHS study participants had high survey adherence at baseline. The majority of participants who returned surveys completed more than 75% of the survey questions across each 3-month period. Adherence decreased over the 12-month follow-up period but remained high compared with that reported in previous mHealth studies. Second, eFHS is embedded in the ongoing prospective FHS, providing us the opportunity to compare surveys collected on the mobile app to questionnaires collected in the research center using standardized protocols. Our data suggest that app-based surveys and research center questionnaires for physical activity, mood, and alcohol intake had moderate to high agreement. Finally, among the subgroup of participants who returned all follow-up surveys, reports of physical activity (PAI) and alcohol consumption were consistent across all 3-month time intervals over the 12-month follow up.

### Comparison With Previous Studies

Although digital and mHealth technologies have great potential to improve health, it is essential to understand engagement challenges with such technologies, as most mHealth studies have observed substantial participant drop offs within a short time period [35]. For example, in the recent Asthma Mobile Health Study [10], more than 40,000 participants downloaded the app, whereas only 7500 participants enrolled in the study and only 175 (2.3%) participants continued to contribute data at the 6-month follow up. Similarly, a study of posttraumatic stress disorder initially had 166,800 participants download the app but only 26,110 (15.7%) users remained after 1 week [36]. The Health eHeart Study is another eCohort that combined the use of social media, smartphones, and wearables to study heart disease. In this groundbreaking cardiovascular study, 86% of consented participants completed at least one survey but 42% of the surveys had missing values [12]. eFHS participants demonstrated considerably higher engagement with a substantial number of participants returning completed surveys at the 12-month follow up (Multimedia Appendix 10). Several reasons may explain the high levels of engagement among eFHS participants. First, the eFHS is embedded in the FHS that consists of loyal participants; the original cohort was followed for more than 70 years, and the present cohort consists of their grandchildren who have been followed in three cycles since the early 2000s. Therefore, the eFHS participants have a long and positive relationship with the study and staff. The smartphone and associated study devices may further connect the participant to the research staff and allow participants to gain insight into their health that could potentially impact engagement [37]. Second, participants received positive notifications, including notifications of thanks and acknowledgement (*“*Thank you for completing your surveys. Your contribution is a vital part in our ongoing research efforts!”), which may have encouraged participation.

Recent studies have tested the equivalence between electronic and paper administration of patient-reported outcome measures [14,15]. These studies focused on acceptability of the different electronic data collection modes, including personal digital assistants using a tablet or personal computer and interactive voice response systems (automated telephone questionnaire), but did not consider mobile apps. Few studies tested the reliability and validity of mobile apps that were specifically designed for special research purposes. The Burden of Obstructive Lung Disease (BOLD) study was conducted to measure the burden of chronic obstructive lung disease; researchers compared the smartphone and paper-based data collection systems in rural Sudan [19]. A new smartphone app of the International Prostate Symptom Score was also developed and tested [18]. These studies demonstrated that smartphone technology worked well compared to paper-based data collection. Nevertheless, validation studies are limited, and more investigation is needed to recommend a mobile app as an effective method of data collection compared to traditional paper-based surveys. Our study demonstrated good agreement between app-based surveys and surveys collected in the research center using standard protocols across a range of measurements, including physical activity, mood, and alcohol intake. Participants reported a slightly higher PAI in the app-based surveys compared to the same questionnaires filled out at the research center. Another study demonstrated equivalence between the paper and smartphone versions of two scales of depression [38], but some electronic items (such as sad mood and trouble concentrating) were lower compared to items on the paper versions even though they had moderate to high consistency. A randomized study was conducted to compare paper versus electronic mode of delivery of a health and social behavior questionnaire [39]. The majority of mode differences were nonsignificant, but participants reported more exercise in the paper survey compared to the electronic survey. This contrasts with our findings in this study as we observed higher PAI scores in app-based surveys. However, smartphone app surveys can be an efficient method to collect cardiovascular risk factor data.

Participants volunteered in the study and there was no mandatary requirement of answering questions in the smartphone app. Adherence varied across different types of surveys. The difficulty of the survey may impact long-term adherence. For example, we observed adherence rates of 40% even after 12 months of follow up for most surveys (n=757), but lower completion rates were obtained for the 12-month medical history update survey (n=28). It is unclear why participants did not complete the medical history update survey. However, this survey was longer than the other surveys in the eFHS app, and compared to other surveys, the medical history update survey contained many open-ended questions that may have required more time and effort to complete. Indeed, unrestricted or open-ended questions have been reported as the major challenge of app-based surveys [19,40].

We conducted a pilot study using two distinct enrollment methods to compare adherence for device use and two internet surveys. The two methods were on-site support (n=101) and remote (n=93) enrollment [41]. The baseline core internet survey consisted of 34 separate parts for self-reported health outcomes, which was completed at home after enrollment and consent. To address the overall study performance, an end-of-study survey was emailed to the participants at study termination. On-site support increased the participation and the initial rate of device use compared to remote support. The pilot study also demonstrated that on-site research center visit was associated with higher adherence to the end-of-study survey. However, on-site research center visit was associated with lower adherence to the internet survey at baseline compared to the remote arm. Thus, the pilot study suggests that in-person contact may not be as important for studies designed to deliver only surveys. We observed higher adherence to the baseline app-based survey in the eFHS than reported for other eCohorts. The eFHS is embedded in the FHS, which consists of loyal participants, potentially leading to higher baseline app-based adherence in the eFHS [37].

A new, enhanced version of the app was implemented in February 2019, which includes an interactive health dashboard to promote participant engagement and facilitate survey adherence. We did not uniformly collect feedback from users, but we are planning to assess usability of the new enhanced version of the app with the Mobile App Rating Scale (MARS) [42] as a separate study.

Insights and responses from participants may help to identify better strategies to improve adherence in mHealth studies [43]. Monitoring and feedback, reminders, goal setting, social support features, and rewards are tools that can be used to improve short- and long-term engagement [44-46]. In particular, the just-in-time adaptive intervention, which aims to provide a proper amount of support at the appropriate time when it is needed, may lead to a change in health behavior [47]. In future work, we plan to establish an advisory panel of eFHS participants to assist with co-design of the app and engagement methods [48].

### Strengths and Limitations

The eFHS is a large community-based cohort designed to study CVD and other risk factors. It is embedded within the ongoing traditional FHS, and the study design provided the opportunity to compare FHS participants who consented vs participants who declined. It also provided the opportunity to compare baseline app surveys and surveys collected in the research center. Importantly, it provided the opportunity to compare eFHS participants to FHS participants who declined enrollment in eFHS to understand the generalizability of the sample.

Our study has several limitations. eFHS included predominantly white, educated, and healthy participants who own smartphones, most of whom resided in the New England region of the United States, thus limiting the ability to generalize the findings from this study to other racial and ethnic groups, individuals with less than high school education, smartphone-naïve, disease-based samples, and other regions/countries. Some participants downloaded the app (n=203) and enrolled in eFHS after January 31, 2019. Since these participants did not have the opportunity to participate in the study for at least 1 year at the time that we began the analysis, we excluded these participants from this study. There were slight differences in the characteristics (Multimedia Appendix 12) of eFHS enrollees who had a greater than 1-year follow-up time vs participants who had a less than 1-year follow-up time (women: 57% vs 50%, married: 75% vs 66%, and median BMI: 27 vs 29), which may not influence the generalizability of the current study. We allowed 90 days to complete baseline app-based surveys, but the participants returned digital surveys within a couple of weeks from the enrollment. The median survey return time varied from 1.35 to 2.40 days and the IQR was about 7 days (Multimedia Appendix 5). Therefore, the survey results relying on self-reported CVD risk factors may not differ according to the time of completing the survey, which would not affect the comparison results of research center measurements (time at enrollment). Moreover, since our study was observational, we cannot exclude residual confounding factors and cannot establish causal relations in our observations of variation in characteristics associated with adherence. A subset of eFHS participants (n=655) were enrolled in a randomized controlled messaging trial (ClinicalTrials.gov NCT03516019) [49] designed to test the impact of personalized notifications on device use. However, the notifications did not impact app-based survey adherence. Recent digital health intervention studies were found to be cost-effective in the management of CVD [50,51]. However, an economic evaluation of digital data was beyond the scope of this study.

### Conclusions

We observed high adherence to baseline surveys with a substantial proportion of participants continuing to complete surveys at the 12-month follow up, indicating that the eFHS app may be a promising tool to collect data. App-based surveys were comparable to the research center–administered questionnaires. Therefore, the eFHS app may serve as a reliable data collection mechanism. Further exploration is needed to understand the reasons for the higher PAI in the app-based surveys compared to the surveys administered at the research center.

## Appendix

Multimedia Appendix 1 (a) eFHS app loading screen and registration steps. (b) The first screen with the list of surveys and steps within the physical activity questionnaire.

Multimedia Appendix 2 Notifications.

Multimedia Appendix 3 eFHS cohort development diagram for the study (app-based survey returns).

Multimedia Appendix 4 Survey description in the eFHS participant protocol.

Multimedia Appendix 5 Survey summary.

Multimedia Appendix 6 Determining the threshold for survey completion.

Multimedia Appendix 7 Number and proportion of eligible eFHS participants completing at least one, all, or none of the periodic app-based surveys within the 3-month window.

Multimedia Appendix 8 Characteristics of participants who returned surveys vs participants who did not return surveys.

Multimedia Appendix 9 Survey return and touch time.

Multimedia Appendix 10 Number of participants who completed 75% of questions of each survey over the 12-month period.

Multimedia Appendix 11 Bland-Altman plots (mobile-based survey measurements vs research center measurements) of Physical Activity Index (PAI), depressive symptoms, and alcohol consumption.

Multimedia Appendix 12 Characteristics of eFHS enrollees by follow-up time.

## Abbreviations

aOR: adjusted odds ratio
CCC: concordance correlation coefficient
CES-D: Center for Epidemiologic-Studies Depression Scale
CVD: cardiovascular disease
eFHS: eCohort Framingham Heart Study
FHS: Framingham Heart Study
mHealth: mobile health
PAI: Physical Activity Index
