# Supplementary material for: Adherence of Mobile App-Based Surveys and Comparison With Traditional Surveys: eCohort Study
Source: J Med Internet Res. 2021 Jan 20;23(1):e24773. doi: 10.2196/24773 (PMC7857942; doi:10.2196/24773)

a) eFHS App Loading Screen and Registration Steps

Welcome Screen **Registration information**

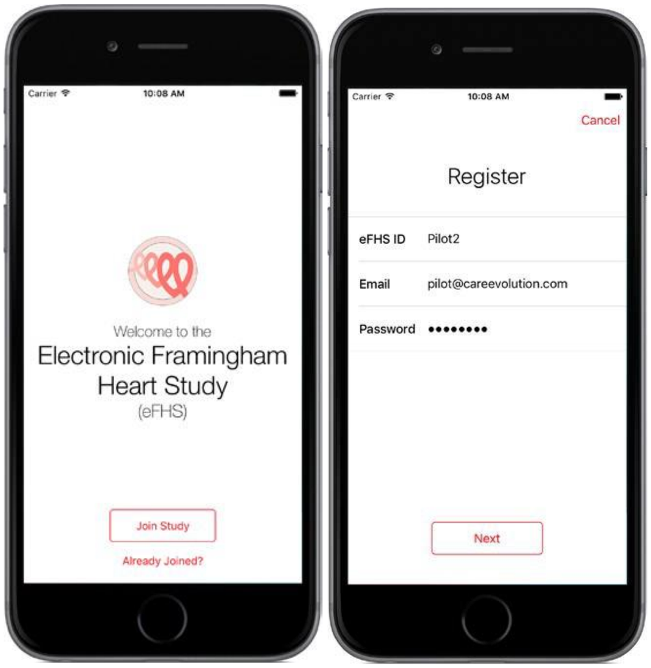

Informed Consents Forms **Notification Permissions** **Registration Complete Screen**

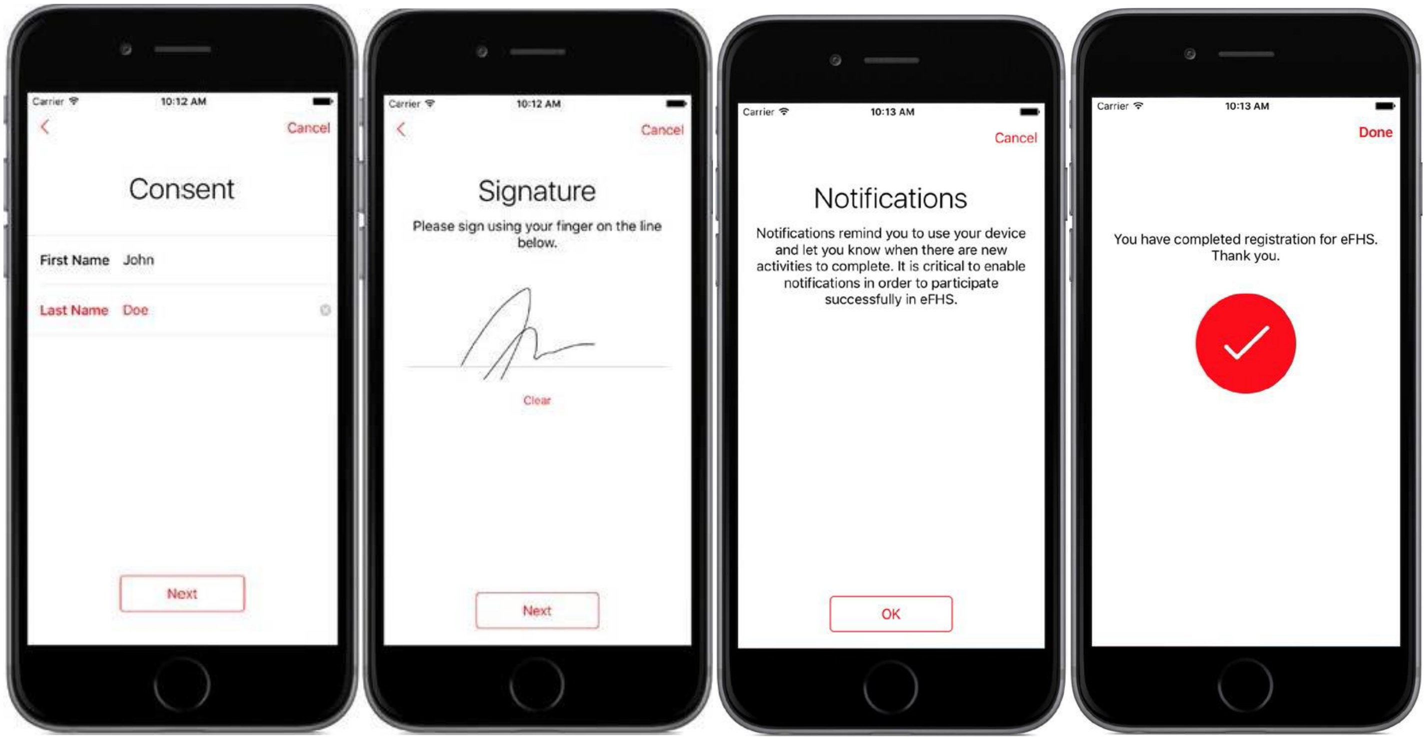

b) The first screen with the list of surveys and steps within the physical activity questionnaire

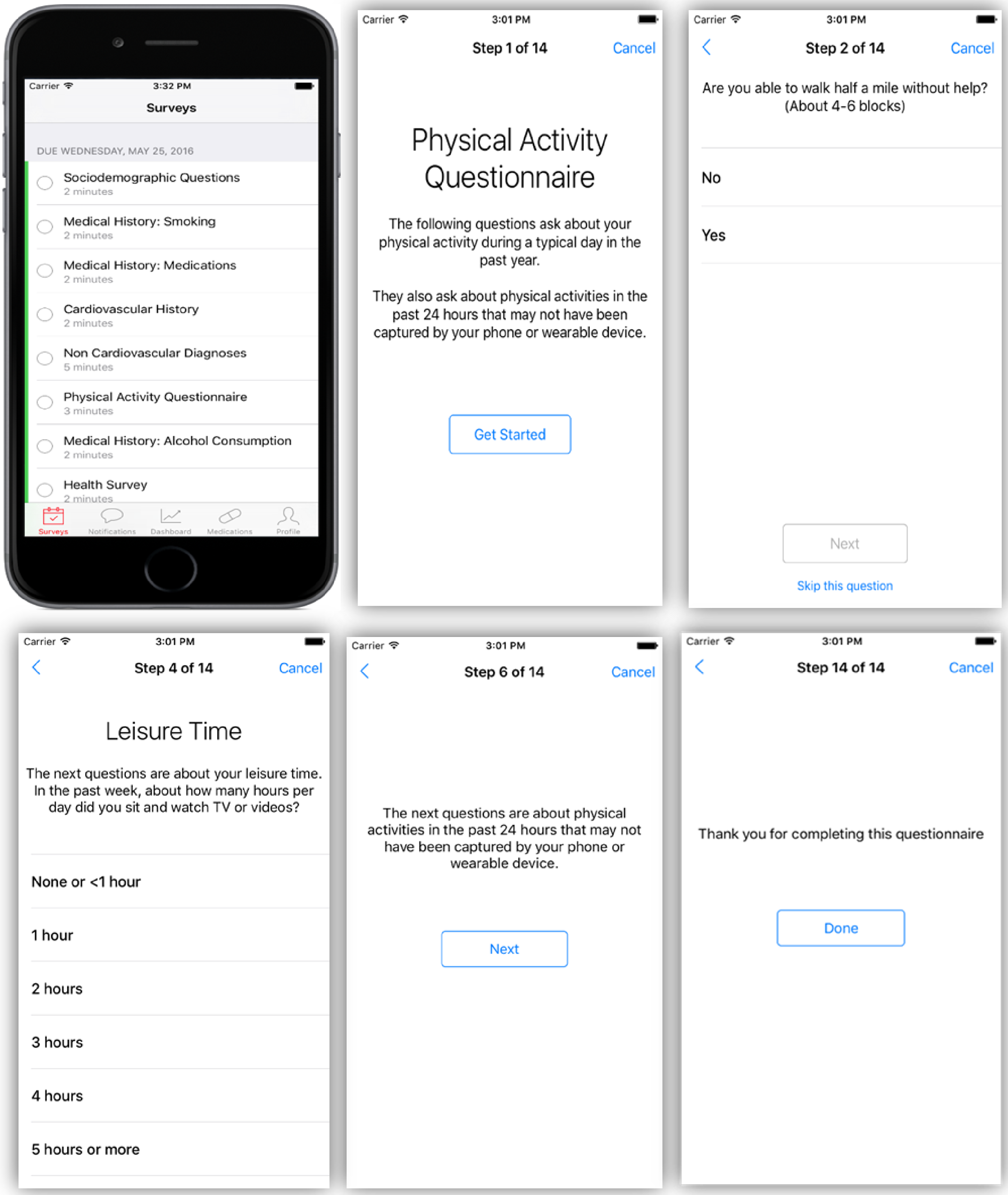

Supplement: Multimedia Appendix 1 [file jmir_v23i1e24773_app1.pdf]
