# Supplementary figures and images for: Adherence of Mobile App-Based Surveys and Comparison With Traditional Surveys: eCohort Study
Source: J Med Internet Res. 2021 Jan 20;23(1):e24773. doi: 10.2196/24773 (PMC7857942; doi:10.2196/24773)

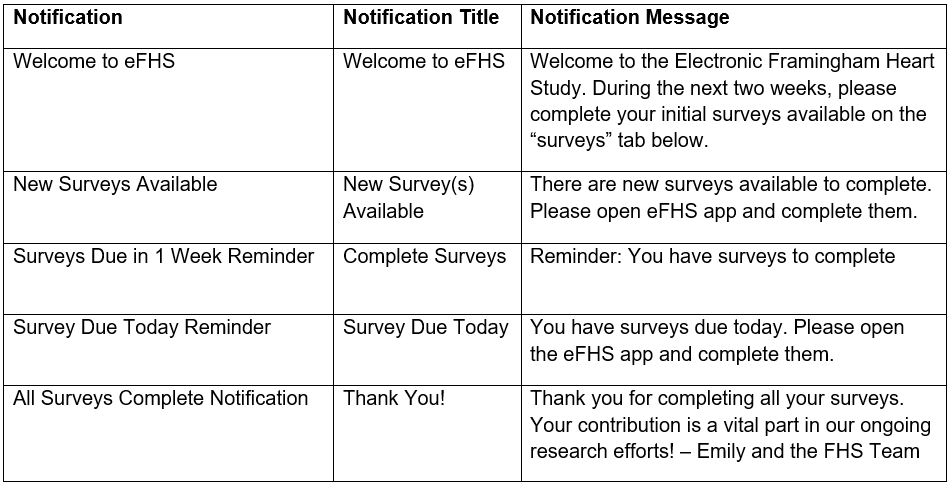

Supplement: Multimedia Appendix 2 [file jmir_v23i1e24773_app2.png]

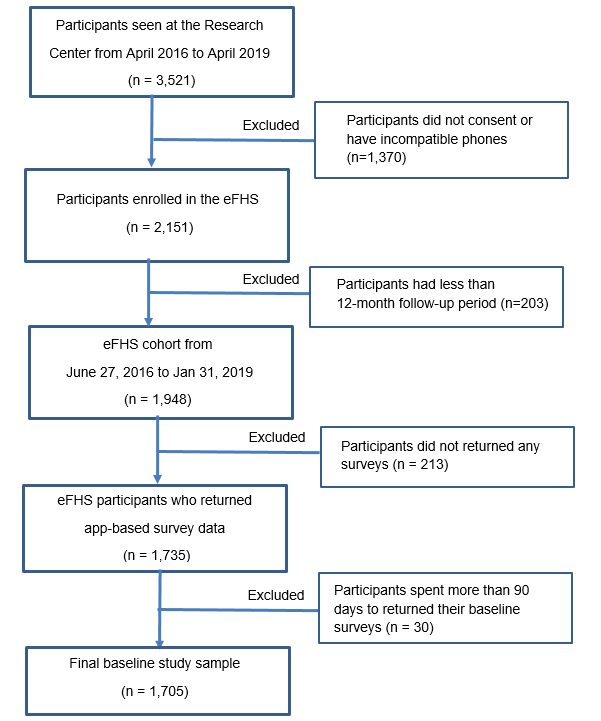

Supplement: Multimedia Appendix 3 [file jmir_v23i1e24773_app3.png]

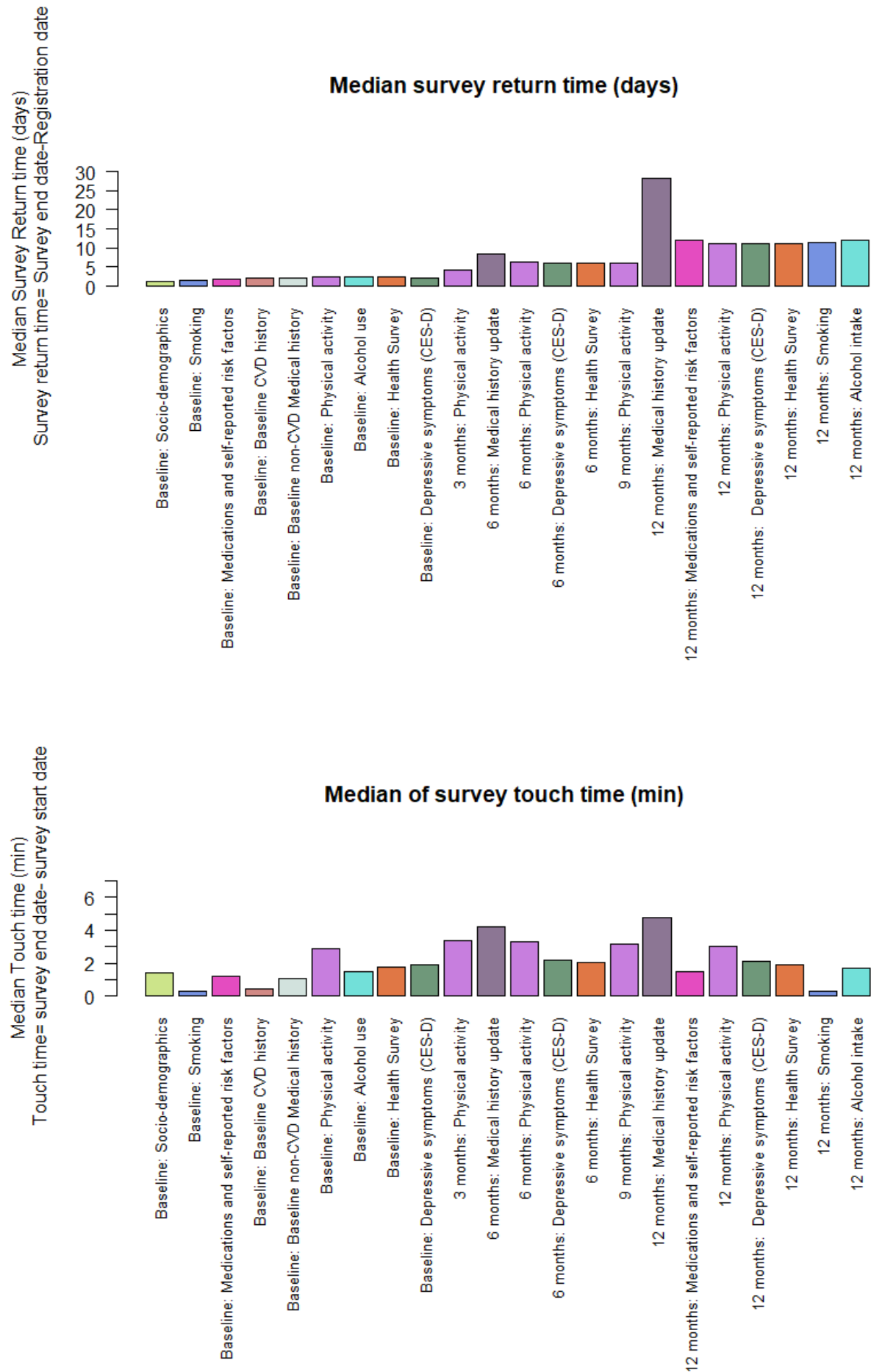

Supplement: Multimedia Appendix 9 [file jmir_v23i1e24773_app9.pdf]

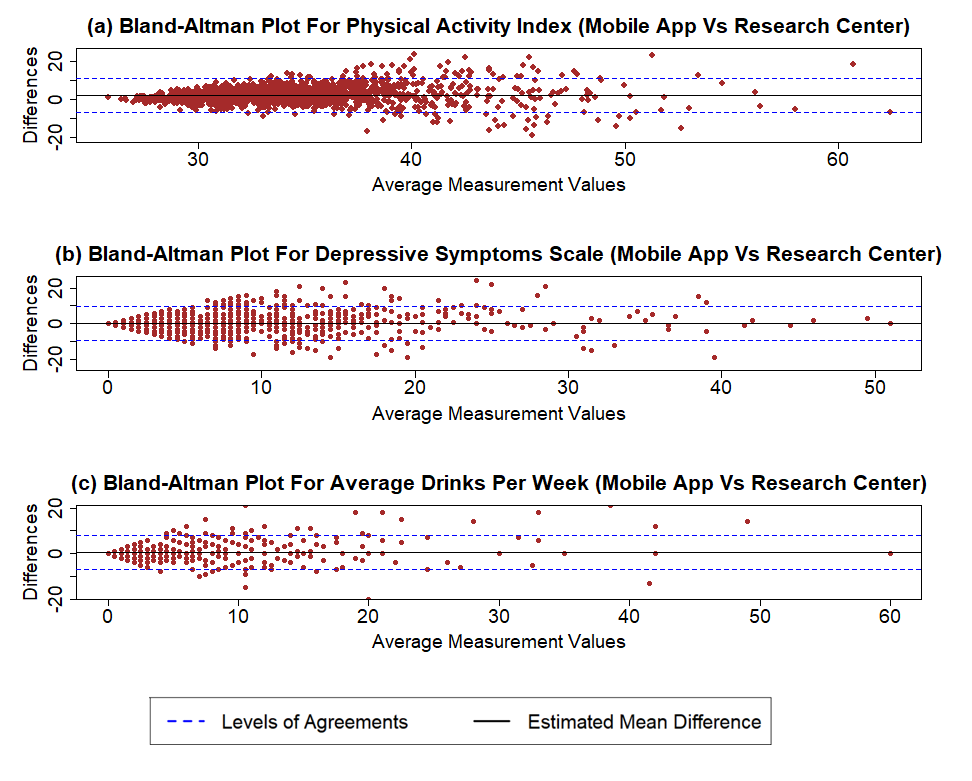

Supplement: Multimedia Appendix 11 [file jmir_v23i1e24773_app11.png]
