# Supplementary material for: Adherence of Mobile App-Based Surveys and Comparison With Traditional Surveys: eCohort Study
Source: J Med Internet Res. 2021 Jan 20;23(1):e24773. doi: 10.2196/24773 (PMC7857942; doi:10.2196/24773)
Supplement: Multimedia Appendix 4 [file jmir_v23i1e24773_app4.pdf]

Type of Survey	Description
Sociodemographic information	The “Sociodemographic Questions” survey asks you about your occupation, marital status, education level, and other sociodemographic factors. This is a baseline survey so it is only available upon first registration: once you have completed it, it will never appear again.
Smoking	The “Medical History: Smoking” survey asks you about your smoking habits and your use of cigarettes, pipes, or e-cigarettes. You will be asked to complete this survey every year.
Medications and self-reported risk factors	The “Medical History: Medications” survey asks you about your medical history, medications, and measurements to evaluate cardiac risk. You will be asked to complete this survey every year.
Baseline CVD history	The “Cardiovascular History” survey asks you about hospitalizations or visits to a doctor for heart or circulatory problems. This is a baseline survey so it is only available upon first registration: once you have completed it, it will never appear again.
Baseline non-CVD medical history	The “Medical History: Non Cardiovascular Diagnoses” survey asks you about hospitalizations or visits to a doctor for non-cardiovascular conditions. This is a baseline survey so it is only available upon first registration: once you have completed it, it will never appear again.
Physical activity	The “Physical Activity Questionnaire” survey asks you about your physical activity during a typical day in the past year. It also asks about physical activities in the past 24 hours that may not have been captured by your wearable device. You will be asked to complete this survey every 3 months.
Alcohol Consumption	The “Medical History: Alcohol Consumption” survey asks you about you alcohol use. You will be asked to complete this survey every year.
Health Survey	The “Health Survey” asks you about how you feel and how well you are able to do your usual activities. You will be asked to complete this survey every 6 months.
Depressive symptoms (CES-D)	The “Mood Survey” asks you about your mood. For each statement, you are asked about your feelings and behaviors during the past week. You will be asked to complete this survey every 6 months.
Medical History update	The “Medical History Update” survey asks you about updates to your contact information and medical history, including hospital admissions, emergency department visits, non-routine office visits, nursing home, or rehabilitation stays. This survey is the only survey that is not available when you first register with the eFHS app. It will become available 6 months after registration, and then every 6 months after it was last completed.
