# Supplementary material for: Adherence of Mobile App-Based Surveys and Comparison With Traditional Surveys: eCohort Study
Source: J Med Internet Res. 2021 Jan 20;23(1):e24773. doi: 10.2196/24773 (PMC7857942; doi:10.2196/24773)
Supplement: Multimedia Appendix 5 [file jmir_v23i1e24773_app5.pdf]

Survey Type	Touch time (min): Survey finish time-Survey Start Time		Survey Return Time (days): Survey Return Date - Survey Deployment Date		Step Time (Seconds)		Time spent for questions (Seconds)	
	Median (min)	IQR (min)	Median (days)	IQR (days)	Median (Sec)	IQR (Sec)	Median (Sec)	IQR (Sec)
**Baseline: Socio-demographics**	1.43	0.94	1.35	5.86	2	1	8	7
**Baseline: Smoking**	0.28	0.98	1.42	6.08	2	1	4	3
**Baseline: Medications and self-reported risk factors**	1.18	1.05	1.95	6.62	1	1	0	2
**Baseline: Baseline CVD history**	0.45	0.56	2.04	6.68	2	1	8	6
**Baseline: Baseline non-CVD Medical history**	1.03	0.88	2.24	6.89	2	2	6	6
**Baseline: Physical activity**	2.85	2.18	2.40	7.00	2	3	3	8
**Baseline: Alcohol use**	1.50	0.88	2.28	6.88	1	1	0	4
**Baseline: Health Survey**	1.73	1.14	2.33	6.90	7	3	7	5
**Baseline: Depressive symptoms (CES-D)**	1.93	1.16	2.26	6.90	2	4	5	3
**3 months: Physical activity**	3.37	2.52	4.36	12.03	2	4	3	9
**6 months: Medical history update**	4.18	3.91	8.52	20.49	2	2	0	6
**6 months: Physical activity**	3.32	2.25	6.20	13.44	2	3	3	8
**6 months: Depressive symptoms (CES-D)**	2.18	1.09	6.09	13.50	3	3	5	4
**6 months: Health Survey**	2.03	1.02	6.09	13.56	2	3	8	5
**9 months: Physical activity**	3.16	2.16	5.99	13.37	2	3	3	8
**12 months: Medical history update**	4.78	5.25	28.22	23.69	2	2.8	0	7
**12 months: Medications and self-reported risk factors**	1.45	1.45	12.06	20.96	1	1	0	2
**12 months: Physical activity**	3.02	2.26	11.00	13.48	2	2	3	8
**12 months: Depressive symptoms (CES-D)**	2.13	1.15	11.06	13.55	2	3	5	4
**12 months: Health Survey**	1.93	1.06	11.01	13.69	2	2	8	5
**12 months: Smoking**	0.32	1.10	11.39	19.08	1	1	4	3
**12 months: Alcohol Consumption**	1.68	0.82	11.97	20.98	1	1	0	4
