# Supplementary material for: Adherence of Mobile App-Based Surveys and Comparison With Traditional Surveys: eCohort Study
Source: J Med Internet Res. 2021 Jan 20;23(1):e24773. doi: 10.2196/24773 (PMC7857942; doi:10.2196/24773)
Supplement: Multimedia Appendix 6 [file jmir_v23i1e24773_app6.pdf]

Multimedia Appendix 6: Determining threshold for survey completion. [a-b]

Survey Type	N	Threshold for Completion of Survey Questions, % (n) at each threshold of completion				
		> 60%	> 70%	> 75%	> 80 %	> 90%
**Baseline: Socio-demographics**	1696	99% (n=1686)	98% (n=1670)	**98% (n=1670)**	95% (n=1622)	84% (n=1420)
**Baseline: Smoking**	1688	100% (n=1688)	100% (n=1688)	**99% (n=1687)**	99% (n=1684)	97% (n=1645)
**Baseline: Medications and self-reported risk factors**	1660	99% (n=1639)	97% (n=1613)	**97% (n=1603)**	95% (n=1590)	92% (n=1525)
**Baseline: Baseline CVD history**	1657	100% (n=1656)	100% (n=1656)	**99% (n=1648)**	99% (n=1648)	96% (n=1593)
**Baseline: Baseline non-CVD Medical history**	1644	100% (n=1643)	99% (n=1628)	**97% (n=1597)**	96% (n=1576)	95% (n=1588)
**Baseline: Physical activity**	1625	95% (n=1550)	95% (n=1545)	**95% (n=1545)**	95% (n=1545)	90% (n=1464)
**Baseline: Alcohol use**	1635	99% (n=1619)	98% (n=1611)	**97% (n=1590)**	95% (n=1553)	92% (n=1509)
**Baseline: Health Survey**	1638	99% (n=1637)	99% (n=1631)	**99% (n=1626)**	99% (n=1626)	98% (n=1619)
**Baseline: Depressive symptoms (CES-D)**	1639	99% (n=1633)	99% (n=1628)	**99% (n=1627)**	99% (n=1625)	98% (n=1607)
**3 months: Physical activity**	1137	98% (n=1114)	97% (n=1110)	**97% (n=1110)**	97% (n=1110)	94% (n=1067)
**6 months: Medical history update**	894	97% (n=871)	94% (n=846)	**90% (n=807)**	98% (n=678)	97% (n=353)
**6 months: Physical activity**	949	99% (n=935)	98% (n=929)	**98% (n=929)**	76% (n=929)	39% (n=872)
**6 months: Depressive symptoms (CES-D)**	963	99% (n=961)	99% (n=960)	**99% (n=959)**	99% (n=958)	98% (n=949)
**6 months: Health Survey**	964	100% (n=963)	99% (n=961)	**99% (n=961)**	99% (n=961)	99% (n=957)
**9 months: Physical activity**	788	99% (n=783)	98% (n=776)	**98% (n=776)**	98% (n=776)	92% (n=731)
**12 months: Medical history update**	33	97% (n=32)	91% (n=30)	**85% (n=28)**	81% (n=27)	33% (n=11)
**12 months: Medications and self-reported risk factors**	692	99% (n=687)	98% (n=675)	**97% (n=674)**	96% (n=665)	93% (n=643)
**12 months: Physical activity**	715	98% (n=706)	98% (n=702)	**98% (n=702)**	98% (n=702)	89% (n=642)
**12 months: Depressive symptoms (CES-D)**	725	100% (n=725)	99% (n=724)	**99% (n=724)**	99% (n=724)	99% (n=718)
**12 months: Health Survey**	721	100% (n=721)	100% (n=721)	**100% (n=721)**	100% (n=721)	99% (n=720)
**12 months: Smoking**	713	100% (n=713)	100% (n=713)	**100% (n=713)**	100% (n=713)	98% (n=701)
**12 months: Alcohol Consumption**	708	99% (n=705)	98% (n=700)	**97% (n=692)**	94% (n=671)	91% (n=648)

[a] Here **N** is the total number of survey returns for each survey type (which includes skipping all steps).
[b] We provide different threshold values for question completion and compute number of participants (n) who complete threshold values of each survey questions (E.g. 1670 participants completed more than 75% of baseline sociodemographic questions)
