# Supplementary material for: Adherence of Mobile App-Based Surveys and Comparison With Traditional Surveys: eCohort Study
Source: J Med Internet Res. 2021 Jan 20;23(1):e24773. doi: 10.2196/24773 (PMC7857942; doi:10.2196/24773)
Supplement: Multimedia Appendix 8 [file jmir_v23i1e24773_app8.pdf]

Multimedia Appendix 8: Characteristics of participants who return surveys vs participants who do not return surveys.[a-d]

Characteristics	No Survey return (n=213)	Survey Returns (n=1735)	Univariable		Multivariable	
			ORs (95% CIs)	*P* value	ORs (95% CIs)	*P* value
**Age, years (mean (SD))**	52.5 (8.1)	52.9 (8.8)	1.00 (0.99-1.02)	0.592	1.01 (0.99-1.02)	0.532
**Female sex, n (%)**	100 (9.0)	1009 (91.0)	1.57(1.18-2.09)	0.002	1.58 (1.18-2.11)	0.002
**Race White, n (%)**	198(10.9)	1615 (89.1)	1.02 (0.56-1.72)	0.946		
**Body mass index, kg/m^2 (mean (SD))**	28.4 (5.2)	28.2 (5.7)	1.00 (0.97-1.02)	0.697		
**Systolic blood pressure, mm Hg (mean (SD))**	119 (14)	119 (14)	1.00 (0.99-1.01)	0.964		
**Diastolic blood pressure, mm Hg**	76 (9)	76 (8)	0.99 (0.98-1.01)	0.561		
**Current smoking, n (%)**	21 (19.4)	87 (80.6)	0.48 (0.30-0.81)	0.004	0.53 (0.32-0.90)	0.015
**Diabetes mellitus, n (%)**	17 (13.9)	105 (86.1)	0.75 (0.45-1.31)	0.282		
**Hypertension, n (%)**	59 (11.5)	452 (88.5)	0.92 (0.67-1.27)	0.613		
**Physical activity index, mean (SD)**	33.8 (6.1)	33.5 (5.0)	0.99 (0.96-1.02)	0.465		
**Highest Education Level Achieved, n (%)**						
Less than or completed high school	27 (14.9)	154 (85.1)	-	-	-	-
Completed some college	54 (11.6)	413 (88.4)	1.34 (0.81-2.19)	0.248	1.29 (0.77-2.12)	0.315
Bachelor's degree	82 (11.1)	659 (88.9)	1.41 (0.87-2.23)	0.152	1.33 (0.81-2.13)	0.239
Graduate or professional degree	49 (8.9)	501 (91.1)	1.79 (1.07-2.94)	0.023	1.65 (0.98-2.74)	0.056
**Married, living as married, living with partner, n (%)**	159 (11.0)	1287 (89.0)	0.94 (0.67-1.31)	0.728		
**Self-Reported Health Excellent, n (%)**	148 (10.5)	1266 (89.5)	1.19 (0.87-1.61)	0.276		
**Employed Full Time, n (%)**	156 (11.5)	1205 (88.5)	0.85 (0.61-1.16)	0.303		

[a] Data reflect enrollment up to January 28, 2019.
[b] Systolic and diastolic blood pressure are from the research examination.
[d] Multivariable model adjusted for age, sex, smoking and highest education level.
