# Supplementary material for: Adherence of Mobile App-Based Surveys and Comparison With Traditional Surveys: eCohort Study
Source: J Med Internet Res. 2021 Jan 20;23(1):e24773. doi: 10.2196/24773 (PMC7857942; doi:10.2196/24773)
Supplement: Multimedia Appendix 10 [file jmir_v23i1e24773_app10.pdf]

Survey Type	Number of participants who completed 75% of questions of each surveys over 12 month period
**Baseline: Socio-demographics**	1670
**Baseline: Smoking**	1687
**Baseline: Medications and self-reported risk factors**	1603
**Baseline: Baseline CVD history**	1648
**Baseline: Baseline non-CVD Medical history**	1597
**Baseline: Physical activity**	1545
**Baseline: Alcohol use**	1590
**Baseline: Health Survey**	1626
**Baseline: Depressive symptoms (CES-D)**	1627
**3 months: Physical activity**	1110
**6 months: Medical history update**	807
**6 months: Physical activity**	929
**6 months: Depressive symptoms (CES-D)**	959
**6 months: Health Survey**	961
**9 months: Physical activity**	776
**12 months: Medical history update**	28
**12 months: Medications and self-reported risk factors**	674
**12 months: Physical activity**	702
**12 months: Depressive symptoms (CES-D)**	724
**12 months: Health Survey**	721
**12 months: Smoking**	713
**12 months: Alcohol Consumption**	692
