# Supplementary material for: Adherence of Mobile App-Based Surveys and Comparison With Traditional Surveys: eCohort Study
Source: J Med Internet Res. 2021 Jan 20;23(1):e24773. doi: 10.2196/24773 (PMC7857942; doi:10.2196/24773)
Supplement: Multimedia Appendix 12 [file jmir_v23i1e24773_app12.pdf]

Multimedia Appendix 12: Characteristics of eFHS enrollees by follow-up time.[a-b]

Variable	eFHS participants who had follow-up time ≥ 12 month (n=1948)	eFHS participants who had follow-up time < 12 month (n=203)	*P*
**Age, years**	52.8 (8.7)	53 (9.5)	0.74
**Female sex, n (%)**	1109 (56.9)	100 (49.9)	0.04
**Race White, n (%)**	1813 (93.1)	184 (90.6)	0.26
**Body mass index, kg/m^2, median [IQR]**	27.3 [7.0]	28.5 [8.8]	0.01
**Systolic blood pressure, mmHg**	119(14)	121 (15)	0.05
**Diastolic blood pressure, mmHg**	76 (8)	75 (9)	0.35
**Current smoking, n (%)**	108 (5.5)	16 (7.9)	0.22
**Diabetes mellitus, n (%)**	122 (6.3)	17 (8.9)	0.22
**Hypertension, n (%)**	511 (26.3)	58 (28.6)	0.53
**Physical activity index, median [IQR]**	32.60 [5.4]	32.9 [6.1]	0.29
**Highest Education Level Achieved, n (%)**			0.48
Less than or completed high school	181 (9.3)	22 (10.8)	
Completed some college	467 (24.1)	55 (27.1)	
Bachelor's degree	741 (38.2)	67 (33.0)	
Graduate or professional degree	550 (28.4)	59 (29.1)	
**Married, living as married, living with partner, n (%)**	1446 (74.7)	131 (65.8)	0.01
**Self-Reported Health Excellent, n (%)**	1414 (72.6)	121 (59.6)	< 0.001
**Employed Full Time, n (%)**	1361 (70.2)	148 (73.6)	0.35

[a] Data reflect enrollment up to January 28, 2019.

[b] Characteristics were presented as mean ± standard deviation for continuous variables (for non-skewed distribution), median and interquartile range (IQR) for skewed distribution, numbers and percentages for nominal variables.
