# Supplementary material for: Adherence of Mobile App-Based Surveys and Comparison With Traditional Surveys: eCohort Study
Source: J Med Internet Res. 2021 Jan 20;23(1):e24773. doi: 10.2196/24773 (PMC7857942; doi:10.2196/24773)
Supplement: Multimedia Appendix 7 [file jmir_v23i1e24773_app7.pdf]

Multimedia Appendix 7: Number and proportion of eligible eFHS participants completing at least one, all, or none of the periodic app-based surveys within the 3-month window.[a-c]

**Variable**	**Baseline Survey (n=1918)**	**3- months Survey (n=1918)**	**6- months Survey (n=1918)**	**9- months Survey (n=1918)**	**12- months Survey (n=1918)**
**Completed at least one survey within relevant period (Survey Adherence)**	1704 (88.80%)	1110 (57.87%)	999 (52.08%)	776 (40.46%)	757 (39.47%)
**Completed all surveys**	1383 (72.10%)	1110 (57.87%)	763 (39.78%)	776 (40.46%)	16 (0.84%)
**Completed No Survey data (do not complete 75% of questions of any given survey)**	214 (11.20%)	808 (40.78%)	919 (47.92%)	1142 (59.54%)	1161 (60.53%)
**Total 1948-30 = 1918**	n=1918	n=1918	n=1918	n=1918	n=1918

[a] Here completing a survey means completing 75% of the questions in the given survey.
[b] Thirty participants were excluded whose first return falls outside the 3 month survey intervals. Thus, total sample size is 1948-30 = 1918.
[c] Among 1705 baseline survey returns, 1704 participants completed at least one baseline survey and completed 75% of questions of the given survey.
